# A combination of probe holder and laser navigation

**DOI:** 10.1186/s40981-018-0157-2

**Published:** 2018-02-21

**Authors:** Yoshimune Osaka, Yoshihisa Morita

**Affiliations:** Department of Anesthesiology, Kawasaki Municipal Hospital, 12-1 Shinkawa Street, Kawasaki-ku, Kawasaki, Kanagawa 210-0013 Japan

To the Editor,

Recently, opportunities for ultrasound-guided peripheral nerve block have been increasing. It is difficult for beginners, and sometimes even for skilled persons, to visualize the needle during the procedure due to the narrow width of the ultrasonic beam. There have been several reports of attempting easy visualization of the needle, for example, with the use of a needle guide [1–3] or laser navigation [4]. However, it is difficult for a single operator, with only two hands available, to perform the different maneuvers, namely, holding a probe, manipulating the needle, and injecting the anesthetic, simultaneously. Herein, we describe a method for undertaking all of the three aforementioned procedures simultaneously using a probe holder, in addition with laser navigation which visualizes easily the needle by adjusting the needle body to the laser (Fig. 1a).

**Fig. 1:**
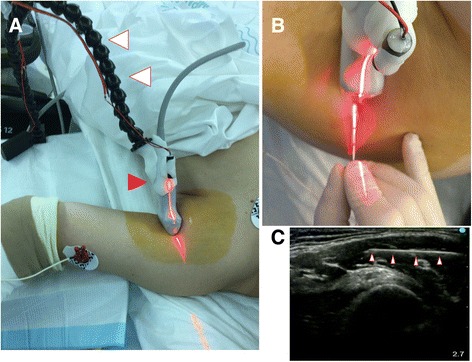
**a** This holder is composed of a flexible arm (white arrows), a holding part, and a laser device (red arrow). **b** With the use of the probe holder, the probe could be held more stable than with the free-hand procedure. The laser navigation assures reliable administration of ultrasound-guided nerve block. **c** The ultrasound image (needle, white arrows)

The fixed probe makes it possible for a person to inject the anesthetic agent and insert a catheter alone. Furthermore, the laser navigation helps to pay attention to the plane of the ultrasound beam. We used the probe holder previously developed by us for a linear probe (S-Nerve HFL50x/15-6^®^, FUJIFILM Medical Co., Ltd., Tokyo, Japan). This device is consisting of three parts, the flexible arm (Flexible arm^®^, PROKIZAI.COM Co., Ltd., Naha, Japan), the probe holder, and the laser module (Line laser module^®^, Denshi Tusho Co. Ltd., Tokyo, Japan). The laser navigation and the probe holder were mounted on the flexible arm (Fig. 1b) using deformable plastic (Moldable Plastic^®^, TFabWorks, Chiba, Japan). Although this combined device is not in commercial, this plastic can be deformed for any type of probes without special tools. It seems to be more stable than using free-hand laser navigation.

The insertion area was cleaned with povidone, and the sterile cover (Cathereeplus™, Nichiban CO. Ltd., Tokyo, Japan) was applied to the probe. Once “the best view” was fixed, the probe holder could keep the position throughout the procedure. We consider this combination as superior to that of the probe holder and the needle guide device because the needle guide is useful only when the insertion site is near the probe. In addition, the laser navigation can predict the cross-section of the ultrasound beam. Despite some clinical limitation, the use of a probe holder combined with laser navigation is very useful for administering ultrasound-guided nerve blocks.

## References

[1] Gupta RK, Lane J, Allen B, Shi Y, Schildcrout JS (2013). Improving needle visualization by novice residents during an in-plane ultrasound nerve block simulation using an in-plane multiangle needle guide. Pain Med.

[2] Whittaker S, Lethbridge G, Kim C, Keon Cohen Z, Ng I (2013). An ultrasound needle insertion guide in a porcine phantom model. Anaesthesia.

[3] Ueshima H, Kitamura A (2015). The use of a needle guide kit improves the stability of ultrasound-guided techniques. J Anesth.

[4] Collins GB, Fanou EM, Young J, Bhogal P (2013). A comparison of free-hand vs laser-guided long-axis ultrasound techniques in novice users. Br J Radiol.

